# Barriers and methods to improve office-based procedural training in a family medicine residency

**DOI:** 10.5116/ijme.573a.364c

**Published:** 2016-05-29

**Authors:** Shannon Langner, Brandy Deffenbacher, John Nagle, Morteza Khodaee

**Affiliations:** 1 University of Colorado School of Medicine, Department of Family Medicine, USA

**Keywords:** Office-based procedural training, family medicine, residency

## Introduction

Family Medicine is a unique field in which the physician is required to be knowledgeable about many different aspects of medicine across all ages, genders, and specialties. This knowledge includes the ability to perform a wide variety of procedures including obstetric, dermatology, gynecology, and sports medicine procedures.^1^^-^^4^ Family Medicine residency programs are faced with the challenge of educating residents about these procedures and ensuring that the residents achieve competency in those skills prior to graduation.^5^^-^^8^ Learning procedural skills is a gradual process that progresses through multiple steps, with the key elements being exposure to and ability to perform the procedure.^9^ During training, there must be a sufficient number of procedures in order for the resident to achieve competency. This paper will discuss a simple intervention that allowed the Family Medicine Center (FMC) to increase the number of procedures performed in the clinic with residents directly involved, thereby, increasing the opportunity for residents to participate and learn these skills.

### Implementing procedure training

#### Needs assessment

An online survey service was used to send a needs assessment to all residents and attending physicians of the FMC. The questions included asking for the types of procedures the physicians performed, importance of performing procedures, reasons for offering procedures in the FMC setting, as well as satisfaction with the number of procedures currently performed in the clinic. For residents there was a question regarding the kind of procedures they thought they were likely to perform upon graduation. Physicians were asked about possible strategies for improving procedure training in the FMC as well as potential barriers to this type of training.   

#### Intervention

An advertising campaign was implemented to inform attending physicians, residents and patients about the procedures offered at the FMC. Information was distributed detailing what types of procedures were offered in clinic and by which attending physician. This list was also given to the referral and call center so that they could schedule appointments with the appropriate physician. Advertisements were placed in each exam room to inform the patients of what types of procedures and visits could be completed in the FMC. In addition to the advertising campaign, adjustments were made to streamline the scheduling of procedures which included simplifying the referral process, educating all healthcare providers in the clinic on the new referral process, scheduling procedures with both an attending physician and a resident, and changing procedure appointment lengths based on the type of procedure being scheduled. 

To evaluate the efficacy of these changes, a retrospective chart audit of two time periods, one before the intervention and one after implementation of the intervention, was completed. The number and type of procedures, as well as the provider performing them, were evaluated. Colorado Multiple Institutional Review Board approved the study.

### What did the providers say?

Out of 32 healthcare providers in our clinic, 28 (88%) completed our survey. The majority of respondents thought that the number of procedures performed and resident involvement in procedures at the clinic was inadequate. Most respondents felt that it was important to provide procedures at our clinic. Most responded that more physician training was needed in order to improve the comfort level of performing procedures. Dermatologic procedures (including punch biopsy, shave biopsy, excisional biopsy) were among the most commonly mentioned procedures physicians were interested in performing in clinic. In addition, residents reported that they were most confident performing dermatologic procedures and were planning to perform them after graduation.

From a scheduling standpoint, respondents felt that the following changes would allow them to perform more procedures:

·       Streamline the procedure referral process to allow for same-day procedures.

·       Designate a specific clinic session for procedures.

·       Increase the amount of 40-60 minute slots in their schedule for procedures.

### What types of procedures were performed?

During the chart audit we reviewed the total number of patients seen, total half day clinic sessions, and total number of procedures performed by residents and attending physicians. There were a total of 177 procedures performed during these two time periods. The most common types of procedures performed were skin procedures (37%), colposcopy (19%), musculoskeletal injections (15%), obstetric ultrasound (11%), and IUD placement (7%). The percentage of the procedures per patient visit did not change; however, there seemed to be relatively more procedures performed by residents. Due to the nature of pilot studies and our small sample size this difference did not reach statistical significance.

## Conclusions

Our needs assessment demonstrated a consistent desire for an increase in the number of procedures performed, the number of physicians performing procedures, and resident access to procedures. The desire for the increase in procedures was expressed equally by both the residents and the faculty. These findings are consistent with prior studies on procedural volume.^2^^,^^10^^,^^11^ The reported reasons for this desire were that it was patient centered and it increased physician satisfaction by increasing the scope and variety of the practice. Prior research has shown that patients expect their medical provider to perform the most common procedures, especially those that can be done in the office.^12^

The needs assessment highlighted that many physicians were not aware of what procedures were offered and who performed them. This finding may help explain why a majority of attending physicians and residents thought the number of procedures performed at the clinic was too low. It is possible that given the lack of knowledge about what was available at the FMC, patients were referred to specialists, contributing to an overall low number of procedures completed. 

Our results demonstrated that residents were performing more procedures after the interventions were implemented. Research has shown that repetition is an important part of learning procedural skills;^9^therefore; this increase in number of procedures completed by our residents is a positive finding. Future changes focusing on the most frequently cited barriers, such as training and the number of physicians offering procedures, could potentially create a larger increase in numbers.

For improvement in resident procedure training, we found that we needed to ensure that healthcare providers, schedulers, and patients were aware of the services we offered as a practice. In addition, our scheduling process needed to be optimized. And, most importantly, resident training needed to be a priority when it came to procedures performed in the FMC.

### Conflicts of interest

The authors declare that they have no conflict of interest.
